# Serum Levels of Migration Inhibitory Factor (MIF) and *In Situ* Expression of MIF and Its Receptor CD74 in Lepromatous Leprosy Patients: A Preliminary Report

**DOI:** 10.3389/fimmu.2018.00246

**Published:** 2018-02-13

**Authors:** Marco Alonso Martinez-Guzman, Anabell Alvarado-Navarro, Vidal Delgado-Rizo, Alejandra Garcia-Orozco, Jorge Arturo Mayorga-Rodríguez, Ana Laura Pereira-Suarez, Mary Fafutis-Morris

**Affiliations:** ^1^Doctorado en Ciencias Biomédicas con Orientación en Inmunología, Departamento de Fisiología, Centro Universitario de Ciencias de la Salud, Universidad de Guadalajara, Guadalajara, Mexico; ^2^Centro de Investigación en Inmunología y Dermatología, Departamento de Fisiología, Centro Universitario de Ciencias de la Salud, Universidad de Guadalajara, Guadalajara, Mexico; ^3^Laboratorio de Inmunología, Departamento de Fisiología, Centro Universitario de Ciencias de la Salud, Universidad de Guadalajara, Guadalajara, Mexico; ^4^Laboratorio de Micología, Instituto Dermatológico de Jalisco “Dr. José Barba Rubio”, Zapopan, Mexico

**Keywords:** leprosy, lepromatous leprosy, skin, serum cytokines, migration inhibitory factor, CD74

## Abstract

Leprosy is a chronic disease caused by *Mycobacterium leprae* that affects the skin and peripheral nerves. It may present as one of two distinct poles: the self-limiting tuberculoid leprosy and the highly infectious lepromatous leprosy (LL) characterized by *M. leprae*-specific absence of cellular immune response. The pro-inflammatory cytokine macrophage migration inhibitory factor (MIF) enhance the bactericide activities of macrophages after interaction with its receptor, CD74. Importantly, MIF also possesses chemoattractant properties, and it is a key factor *in situ* for the activation of macrophages and in blood to promote leukocytes migration. MIF-mediated activation of macrophages is a key process for the elimination of pathogens such as *Mycobacterium tuberculosis*; however, its participation for the clearance of *M. leprae* is unclear. The aim of this study was to evaluate the serum levels of MIF as well as MIF and CD74 expression in skin lesions of LL and compare it with healthy skin (HSk) taken from subjects attending to dermatological consult. Samples of serum and skin biopsies were taken from 39 LL patients and compared with 36 serum samples of healthy subjects (HS) and 10 biopsies of HSk. Serum samples were analyzed by ELISA and skin biopsies by immunohistochemistry (IHC). IHC smears were observed in 12 100× microscopic fields, in which percentage of stained cells and staining intensity were evaluated. Both variables were used to calculate a semi-quantitative expression score that ranged from 0 to 3+. We found no differences in MIF levels between LL patients and HS in sera. In addition, MIF was observed in over 75% of cells with high intensity in the skin of patients and HSk. Although we found no differences in MIF expression between the groups, a CD74 score statistically higher was found in LL skin than HSk (*p* < 0.001); this was the result of a higher percentage of cells positive for CD74 (*p* < 0.001). As a conclusion, we found that CD74-positive cells are intensely recruited to the skin with LL lesions. In this manner, MIF signaling may be enhanced in the skin of LL patients due to increased expression of its receptor, but further studies are required.

## Introduction

Leprosy is a chronic infectious disease caused by *Mycobacterium leprae*. The disease in patients may develop as one of two poles that differ clinically and immunologically between each other. Few lesions in the body and an active immune response mediated by cells characterize the tuberculoid leprosy (TT) pole; on the other hand, patients who develop lepromatous leprosy (LL) show numerous erythematous macules around the body coupled with *M. leprae*-specific anergy and disrupted immune response. The reasons for developing either pole are still unknown, but the environment and host genetics are probable factors that mediate such polarization (1). As a consequence, the ratio of LL to TT patients varies around the world, and it can be as high as 3:1 in countries like Mexico, where TT cases are scarce (2).

In LL patients, *M. leprae* proliferates in the skin and peripheral nerves (1). Histologically, skin lesions of LL patients are formed by diffuse infiltration of macrophages, lymphocytes, and plasma cells in the dermis, along with a dysfunctional epidermis. Anti-inflammatory cytokines, such as IL-10 and TGF-β, are highly expressed in these lesions when compared with TT lesions (3). As a consequence of the immunological imbalance, macrophages get infested by abundant bacilli and become foamy macrophages (4). These macrophages seem unable to kill *M. leprae* but serve as a reservoir instead and release additional anti-inflammatory mediators and reduce their production of key activator cytokines, namely TNF-α and IL-1β (5).

Macrophage migration inhibition factor (MIF) is a constitutively released cytokine that is able to subvert the anti-inflammatory activities of glucocorticoids and also initiates intense immune reactions by stimulating macrophages toward an inflammatory profile that involves productions of ROS, increases in the expression of TLR4, and diminishes cell susceptibility to apoptosis (6, 7). MIF participates not only in skin homeostasis by regulating the differentiation and proliferation of keratinocytes (8) but also in skin disorders by enhancing the activity of inflammatory macrophages (9). To exert its biological activities, MIF needs to interact with its receptor CD74, a molecule with a wide array of described functions in immune cells (10). In addition, CD74 may form a complex with chemokine receptors and lead cells to a process of MIF-mediated migration (11).

Only few studies analyze the expression of both MIF and CD74 in infectious diseases, even though MIF has proved to be an important mediator against several infections, especially those caused by *Mycobacterium tuberculosis* (12) and *Leishmania major* through a mechanism dependent on TNF-α and reactive nitrogen intermediates (13). In addition, the early stimulation of CD74 triggers the MAPK and PI3K pathways that lead to proliferation and recruitment of immune cells, thus starting the inflammation in injured tissues (7). However, the specific role of MIF/CD74 interaction in the context of skin diseases has been poorly studied. Due to its relevance in the inflammatory process, it is interesting to know whether the MIF/CD74 axis is implicated in the immunopathology of LL. We previously reported that the susceptibility to develop LL in Western Mexico is associated to the alleles of the STR-794 CATT_5–8_ polymorphism of the *MIF* gene linked to higher expression of MIF (14). This paradoxical observation relates the inflammatory cytokine MIF with the anti-inflammatory LL pole and is suggestive of an important role for MIF in this spectrum of leprosy. Thus, we are interested to study the levels of MIF in the serum of LL patients as well as the expression of both MIF and CD74 in the skin lesions of LL patients.

## Materials and Methods

### Subjects and Samples

Patients for this study were diagnosed by Dermatologists working in the Instituto Dermatológico de Jalisco “Dr. José Barba Rubio” according to their clinic, bacilloscopic, and histopathologic characteristics. Due to the low prevalence of TT in Mexico (2), only patients diagnosed as LL and borderline lepromatous in the absence of reactional episodes were included in the study. Patients who presented chronic, inflammatory, and dermatological conditions were excluded. Serum samples from patients were requested upon diagnosis confirmation. Healthy subjects (HS) of similar age and gender characteristics were asked for blood donation to compare MIF serum levels. In total, serum of 39 patients and 36 HS were considered for determination of MIF. The demographic and disease characteristics of participants are shown in Table 1.

**Table 1:** Demographic and disease characteristics of participants.

	HS	LL
Age (years)	40.5	57.5
Gender		
Male (*n*, %)	16 (44.4)	20 (51.3)
Female (*n*, %)	20 (55.6)	19 (48.7)
Duration of disease (years)	14.3	
Baciloscopic index (*n*, %)		
0		1 (2.5)
1		4 (10.3)
2		2 (5.1)
3		5 (12.8)
4		9 (23.1)
5		9 (23.1)
6		9 (23.1)

Tissue samples of 39 patients were obtained for immunohistochemistry (IHC) assays from the biopsies taken for their diagnostics; in addition, 10 tissues of healthy skin (HSk) were obtained from the repertoire of the Institute. Samples were embedded in paraffin for preservation and cut into 3-µm sections for mounting onto precharged slides.

### Serum Quantification of MIF

Migration inhibitory factor quantification was performed by the ELISA kit for “Human MIF Immunoassay” (Cat. No. DMF00B; R&D, MN, USA) following the manufacturer’s indications. Briefly, samples were diluted 10-fold with the kit Calibrator Diluent and 50 µl of the mix were added to the microplate wells, followed by 2-h incubation. Wells were washed and the detection antibody was incubated for 2 h. Wells were washed and the color was developed by 30-min incubation with Substrate Solution in the dark. Finally, stop solution was added and optical density was measured at 450 nm and corrected at 570 nm. The concentration of MIF was calculated interpolating the optical density of samples with a multiparametric curve generated with the MIF standard included in the kit.

### *In Situ* Characterization of MIF and CD74

We analyzed the expression of MIF and CD74 in skin biopsy samples. Slices of tissue samples were incubated at 60°C for 10 min and deparaffinized on xylene bath for 20 min. Samples were then rehydrated through a series solutions of decreasing concentrations of ethanol. Antigens were retrieved in a bath of sodium citrate solution 10 mM at 95°C for 10 min followed by cooling in a citrate cold solution. Non-specific binding to proteins was blocked by incubation with bovine fetal serum 10% by 30 min; endogenous peroxidase activity of samples was blocked by incubation with H_2_O_2_ 3% solution by 30 min. Afterward, sections of each biopsy were incubated overnight at 4°C with one of the following primary antibodies: anti-MIF FL-115 (Cat# sc-20121 RRID:AB_648587) 2 µg/ml or anti-CD74 FL-296 (Cat# sc-20082 RRID:AB_2075501) from Santa Cruz Biotechnology, Inc., TX, USA. The detection of primary antibodies will be performed using the polymer conjugated to secondary antibodies from Dako EnVision™ + Dual Link System-HRP (Dako Agilent Technologies, Denmark). Finally, sections were counterstained with hematoxylin. The presence of antigens were analyzed in four random 40× fields using the semi-quantitative algorithm of Li et al., which took into account both the intensity of staining and the percentage of stained cells (15). Briefly, percentage of stained cells was transformed into histological index I (HI I) as follows: Neg (0+) ≤ 5% of stained cells; “+” (1+) = 6–25%, “++” (2+) = 26–50%, “+++” (3+) = 51–75%; and “++++” (4+) > 76%. Similarly, a second histological index (HI II) for staining intensity was calculated as: Neg (0+) = no staining, “+” (1+) = low intensity, “++” (2+) = moderate intensity, “+++” (3+) = high intensity. Finally, HI I and HI II were multiplied and ranked as an expression score of Neg if the product of (HI I)*(HI II) was between 0 and 1; “+” (1+) if the product was between 2 and 4; “++” (2+) if the product was between 5 and 8; and “+++” (3+) if the product was between 9 and 12.

### Statistical Analysis

Qualitative variables of the study were expressed as frequencies ± SD and quantitative variables were expressed as mean values ± SD. Statistical analyses were performed on IBM SPSS Statistics ver. 20. Differences in MIF levels between groups, as well as differences of expression score, percentage of stained cells, and staining intensity for MIF and CD74 were analyzed using Mann–Whitney *U* test. In addition, we studied whether there exists correlation between the bacillary index of LL patients, the soluble levels of MIF as well as the *in situ* expression of MIF and CD74 using the non-parametric one-tailed Rho spearmen correlation coefficient. Statistical analysis resulting with *p* values <0.05 were considered as significant.

### Ethical Consideration

The study was designed in agreement with the Declaration of Helsinki (16). All participants were informed about the goals of the study and provided written agreement. This study was approved by the ethical committee *Comité de Investigación y Bioseguridad del Centro Universitario de Ciencias de la Salud* of the University of Guadalajara (No. CI-02515).

## Results

### Serum Levels of MIF

We quantified the serum levels of MIF in 39 LL patients (44.49 ± 20.21 ng/ml) and 36 HS (53.35 ± 34.13 ng/ml). However, we found no significant differences regarding the levels of MIF between groups. Also, we did not find significant correlation between the bacillary index and serum MIF levels (*r* = 0.272, *p* = 0.154).

### MIF-Related Markers in skin

We analyzed the expression of MIF and CD74 in skin biopsies of 39 LL patients as well as 10 samples of HSk.

**Figure 1:**
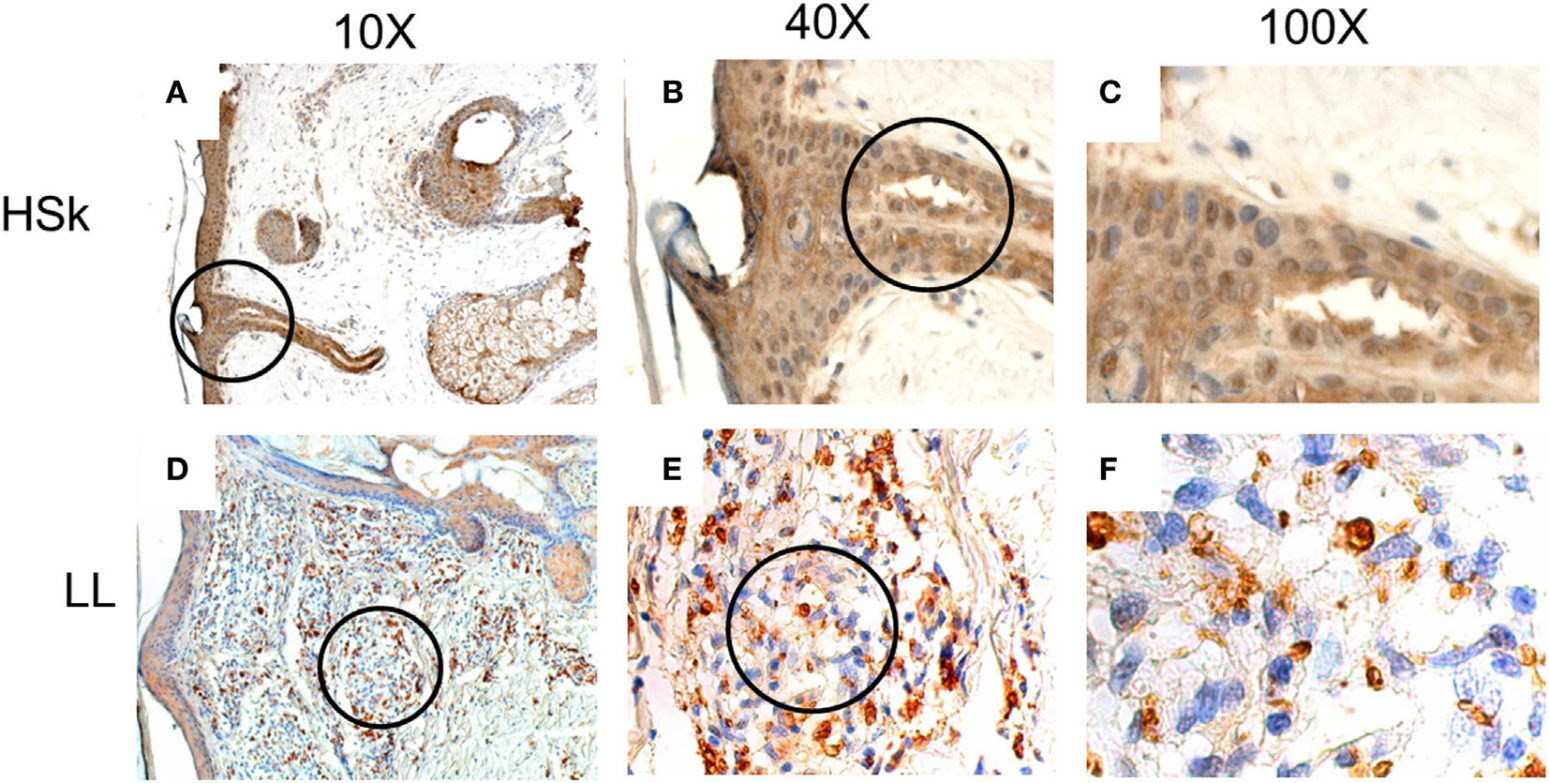
*In situ* expression of migration inhibitory factor (MIF) in healthy skin (HSk) and lepromatous leprosy (LL) skin biopsy. Photographs of MIF-directed immunohistochemistry assays on HSk and skin lesions of LL patients on progressive magnifications are displayed. **(A–C)** represent the staining of MIF on HSk at 10×, 40×, and 100×, respectively. **(D–F)** show staining of MIF on LL skin at 10×, 40×, and 100×. The magnified fields are shown within circles. MIF expression is intense on epidermis and annexes of both HSk and LL. In dermis, its expression also seems to be constitutive. MIF is expressed mostly in cytoplasm and can also be found in some nuclei.

We found that MIF is highly expressed in the epithelia of both patients and HSk (Figure 1). There exist also several MIF^+^ cells in the dermis in both groups. We observed that MIF is mainly expressed in the cytoplasm but it can also be found in some nuclei. We found no significant differences in the expression score between both groups (Table 2). Most of the participants presented 1+ or 2+ of MIF expression score in skin samples. To better understand this index, we analyzed the percentage and intensity index. We found that most of the LL skin samples could be classified as 1+ (37.2%) or 2+ (34.9%); similarly, most of HSk fell into 1+ (30%) and 2+ (40%) categories. Interestingly, the staining of MIF in cells was a little stronger in HSk than in LL samples, but no significant differences were found. In addition, MIF expression score was not correlated to the bacillary index of patients (*r* = 0.190, *p* = 0.153).

**Table 2:** Ranking of lepromatous leprosy (LL) patients and healthy skin (HSk) according to their score of migration inhibitory factor (MIF) staining.

Score							

MIF	Negative	+	++	+++		Total	*p* = 0.990
LL (*n* = 39)	16.3%	37.2%	34.9%	11.6%		100%	
HSk (*n* = 10)	20%	30%	40%	10%		100%	

**Percentage of stained cells**							

	**Negative**	**+**	**++**	**+++**	**++++**	**Total**	***p* = 0.550**

LL (*n* = 39)	14.0%	16.3%	32.5%	23.2%	14.0%	100%	
HSk (*n* = 10)	10%	10%	30%	40%	10%	100%	

**Staining intensity**							

	**Negative**	**+**	**++**	**+++**		**Total**	***p* = 0.499**

LL (*n* = 39)	11.6%	14.0%	44.2%	30.2%		100%	
HSk (*n* = 10)	0%	40%	40%	20%		100%	

*Statistical analysis using two-sided Mann–Whitney U test, where p < 0.05 values were considered as significant*.

**Figure 2:**
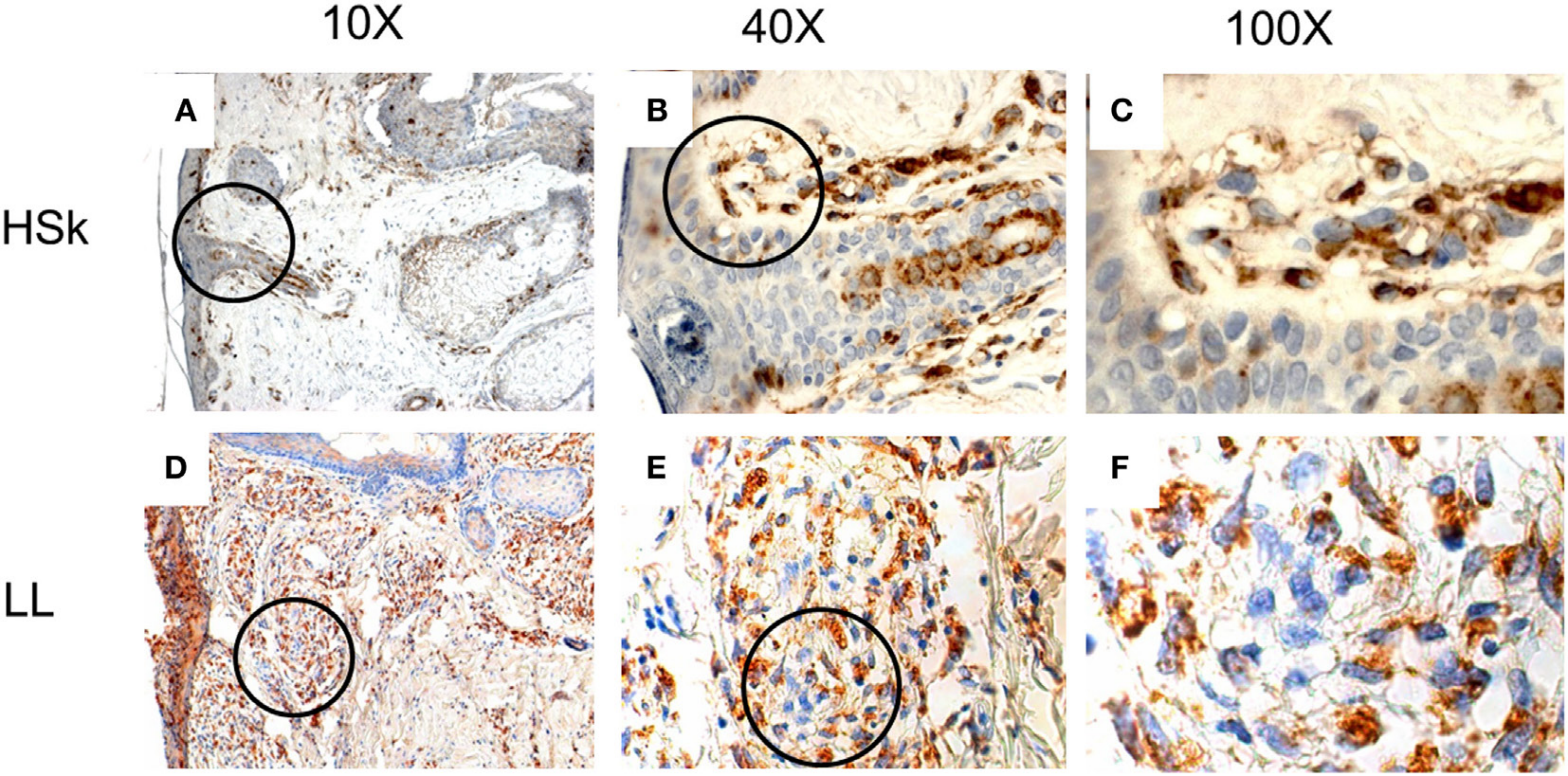
*In situ* expression of CD74 in healthy skin (HSk) and lepromatous leprosy (LL) skin biopsy. Photographs of CD74-directed immunohistochemistry assays on HSk and skin lesions of LL patients on progressive magnifications are displayed. **(A–C)** represent the staining of CD74 on HSk at 10×, 40×, and 100×, respectively. **(D–F)** show staining of CD74 on LL skin at 10×, 40×, and 100×, respectively. The magnified fields are shown within circles. CD74 is scarcely expressed in epidermis and annexes. It is expressed in the dermis, where a major infiltrate can be observed in LL skin than HSk. Paradoxically, it seems to be expressed mostly in cytoplasm rather than the plasma membrane.

CD74 is expressed in the dermis, but its epidermal expression is scarce (Figure 2). Although it is expressed in the membrane of some cells, it is mostly expressed in the cytoplasm. Interestingly, we found that its expression score is significantly higher in LL skin than HSk (*p* < 0.001), since 0% of HSk could be classified as 2+ or 3+, whereas 32.6 and 23.3% of LL was classified as 2+ and 3+, respectively (Table 3). We analyzed whether this difference was due to an increased infiltrate of CD74^+^ cells, a higher staining intensity or both. We found that none of the HSk presented an infiltrate of over 1+ (equivalent to 25% of cells), whereas 27.9% LL skin samples presented 2+, 46.5% presented 3+, and 4.7% of LL samples presented 4+. However, the intensity of CD74 staining was not different between groups; notably, CD74 presented high intensity (3+) in up to 40% of HSk and 39.5% of LL. Finally, CD74 expression score was not correlated to the bacillary index of patients (*r* = 0.040, *p* = 0.416).

**Table 3:** Ranking of lepromatous leprosy (LL) patients and healthy skin (HSk) according to their score of CD74 staining.

Score							
CD74	Negative	+	++	+++		Total	*p* < 0.001
LL (*n* = 39)	7.0%	37.1%	32.6%	23.3%		100%	
HSk (*n* = 10)	40%	60%	0%	0%		100%	

**Percentage of stained cells**							

	**Negative**	**+**	**++**	**+++**	**++++**	**Total**	***p* < 0.000**

LL (*n* = 39)	4.7%	16.3%	27.9%	46.5%	4.7%	100%	
HSk (*n* = 10)	30%	70%	0%	0%	0%	100%	

**Staining intensity**							

	**Negative**	**+**	**++**	**+++**		**Total**	***p* = 0.422**

LL (*n* = 39)	4.7%	11.6%	44.2%	39.5%		100%	
HSk (*n* = 10)	20%	20%	20%	40%		100%	

*Statistical analysis using two-sided Mann–Whitney U test, where p < 0.05 values were considered as significant*.

## Discussion

The immune response has been well characterized in the advanced forms of leprosy, but there are still several gaps where research is required. One of the main differences between the two poles is the presence of inflammatory cells and markers in TT, meanwhile, anti-inflammatory conditions are rather present in LL patients (3); however, it is not clear what mechanisms lead to such polarization. We previously reported that *MIF* polymorphisms in the promoter region are associated with LL in Western Mexico population (14). Thus, in this work, we investigated the serum levels of MIF in LL patients. Despite the genetic association, we found no differences in the serum levels of MIF between LL patients and HS. Recently, Bansal et al. measured MIF levels in leprosy patients and they also found that the MIF levels in serum are similar between healthy controls and patients presenting LL, borderline lepromatous, or borderline tuberculoid (17).

To obtain more information about MIF role in leprosy, we examined skin biopsies by IHC to inquire about its cellular and tissular localization. As in serum, we found that the expression of MIF is not different between skin lesions of LL patients and HSk, despite the elevated expression of IL-4, IL-10, and TGF-β reported in LL skin lesions (18). Therefore, we may postulate that anti-inflammatory mediators do not reduce the expression of MIF, although they may interfere with MIF capacity to enhance the bactericidal activities of macrophages, which is an intrinsic activity of MIF (6). Since MIF can be stored within the cytoplasm (19), our results most likely reflect the constitutive expression of MIF (20). Noteworthy, MIF presence is crucial for cell homeostasis due to its multiple activities. It may be secreted from the central nervous system to regulate cell proliferation (6), and it also promotes glucose intake by increasing the expression of GLUT4 (21). Nevertheless, MIF functions may vary according to other microenvironment components and the cell that it is targeting. For example, MIF expression is increased in inflammatory skin diseases such as alopecia areata and skin tumors (22, 23) and MIF orthologs are produced by *Leishmania* spp. to promote the survival on parasite-infected macrophages (24).

Since MIF expression is not different between LL skin and HSk, we measured CD74 expression to determinate if it is involved in leprosy lesions. Paradoxically, we found that its expression is significantly higher in LL skin than HSk. However, the elevated expression of CD74 does not correlate with the anti-inflammatory environment of LL lesions. In addition to being MIF receptor, CD74 excerpts several functions that include regulation of vesicular transport, dendritic cells migration, and protection of proteins as a chaperone (11). Noteworthy, we found that CD74 is expressed mostly in the cytoplasm and that few cells actually expressed CD74 on the plasma membrane, suggesting that in LL, CD74 is not acting as a receptor for MIF, whereas its overexpression could contribute to the lack of response toward *M. leprae*. Arguably, the transport of antigens on MHC-II toward the plasma membrane of monocytes could be altered. Indeed, the expression of both MHC-I and MHC-II are reduced in dendritic cells infected with *M. leprae* in a dose-dependent manner (25). Moreover, Lee et al. have described that the expression of leukocyte Ig-like receptor A2 (LILRA2) is decreased in TT compared with LL, where it reduced the capacity of monocytes to present leprosy antigens on MHC-II to T cells, although antigen processing was not disrupted (26). Given that CD74 is a chaperone for MHC-II (27) and that it is involved in vesicle transport (11), cytoplasmic CD74 in LL could arrest the movement of antigens-loaded MHC-II toward the cell surface in an LILRA2-mediated mechanism.

Given that 80% of new cases of leprosy Mexico are multibacillary cases (2), our observations of MIF and CD74 are limited to LL patients without treatment. It is important to pursue paucibacillary cases to better detail the role of MIF in leprosy; in particular, the study of MIF in indeterminate leprosy results of particular interest due to the functions of MIF in innate immunity (6, 28). In addition, its participation in reactional episodes has been highlighted by Bansal et al., whose group found increased serum concentration of MIF in erythema nodosum leprosum (17). Further description of MIF and CD74, as well as the possibly involved signaling pathways, namely, PI3K, MAPK could yield valuable insight into leprosy immunopathology.

In summary, we have found that the expression of MIF in LL patients is similar to HS both in serum and in skin. However, the expression of CD74 is significantly increased in the skin lesions of LL patients, although its participation in the physiopathology leprosy remains unclear. Further studies in indeterminate leprosy and paucibacillary leprosy, as well as other infectious and inflammatory diseases of skin, are required to describe the participation of MIF/CD74 in the immune response against leprosy in the skin. In addition, their activities in the systemic response should also be explored. It is important to determine the molecules to which CD74 is binding to further understand the regulation of antigen presentation in the LL skin. In addition, the characterization of MIF^+^ and CD74^+^ cells could provide further insight into the immune microenvironment of skin lesions.

## Ethics Statement

This study was carried out in accordance with the recommendations of Comité de Investigación y Bioseguridad del Centro Universiario de Ciencias de la Salud of the University of Guadalajara (No. CI-02515) with written informed consent from all subjects. All subjects gave written informed consent in accordance with the Declaration of Helsinki. The protocol was approved by the Comité de Investigación y Bioseguridad del Centro Universitario de Ciencias de la Salud of the University of Guadalajara (No. CI-02515).

## Author Contributions

MM-G aided in ELISA assays and performed IHC identification of MIF and CD74; AA-N directed and designed the IHC assays; VD-R directed the ELISA assays; AG-O performed the ELISA assays; JM-R and AP-S performed the statistical analyses; MF-M is responsible for conception of the work and supervised the progress at all times. All authors participated in drafting, revising, and approval of the manuscript.

## Conflict of Interest Statement

The authors declare that the research was conducted in the absence of any commercial or financial relationships that could be construed as a potential conflict of interest.

## References

[1] Lastória JC Leprosy: review of the epidemiological, clinical, and etiopathogenic aspects – part 1. An Bras Dermatol (2014) 89:205–18. 10.1590/abd1806-4841.20142450 24770495 PMC4008049

[2] World Health Organization. Weekly Epidemiological Record Relevé Épidémiologique Hebdomadaire. (2017). Available from: http://apps.who.int/iris/bitstream/10665/255611/1/WER9222.pdf?ua=1

[3] de Sousa JR de Sousa RPM de Souza Aarão TL Dias LB Carneiro FRO Fuzii HT In situ expression of M2 macrophage subpopulation in leprosy skin lesions. Acta Trop (2016) 157:108–14. 10.1016/j.actatropica.2016.01.008 26827741

[4] Massone C Belachew WA Schettini A. Histopathology of the lepromatous skin biopsy. Clin Dermatol (2015) 33:38–45. 10.1016/j.clindermatol.2014.10.003 25432809

[5] Yang D Shui T Miranda JW Gilson DJ Song Z Chen J *Mycobacterium leprae*-infected macrophages preferentially primed regulatory T cell responses and was associated with lepromatous leprosy. PLoS Negl Trop Dis (2016) 10(1):e0004335. 10.1371/journal.pntd.0004335 26751388 PMC4713426

[6] Calandra T Roger T. Macrophage migration inhibitory factor: a regulator of innate immunity. Nat Rev Immunol (2003) 3:791–800. 10.1038/nri1200 14502271 PMC7097468

[7] Tillmann S Bernhagen J Noels H. Arrest functions of the MIF ligand/receptor axes in atherogenesis. Front Immunol (2013) 4:115. 10.3389/fimmu.2013.00115 23720662 PMC3655399

[8] Ashcroft GS Mills SJ Lei K Gibbons L Jeong MJ Taniguchi M Estrogen modulates cutaneous wound healing by downregulating macrophage migration inhibitory factor. J Clin Invest (2003) 111:1309. 10.1172/JCI200316288.Introduction 12727922 PMC154440

[9] Steinhoff M Meinhardt A Steinhoff A Gemsa D. Evidence for a role of macrophage migration inhibitory factor in psoriatic skin disease. Br J Dermatol (1999) 141(6):1061–6. 10606853 10.1046/j.1365-2133.1999.03206.x

[10] Leng L Metz CN Fang Y Xu J Donnelly S Baugh J MIF signal transduction initiated by binding to CD74. J Exp Med (2003) 197:1467–76. 10.1084/jem.20030286 12782713 PMC2193907

[11] Schröder B The multifaceted roles of the invariant chain CD74 – more than just a chaperone. Biochim Biophys Acta (2016) 1863:1269–81. 10.1016/j.bbamcr.2016.03.026 27033518

[12] Das R Koo M Hoon B Jacob ST Subbian S Yao J Macrophage migration inhibitory factor (MIF) is a critical mediator of the innate immune response to *Mycobacterium tuberculosis*. Proc Natl Acad Sci U S A (2013) 110(32):E2997–3006. 10.1073/pnas.1301128110 23882081 PMC3740876

[13] Juttner S Metz CN Ro M Bucala R Migration inhibitory factor induces killing of *Leishmania major* by macrophages: dependence on reactive nitrogen intermediates and endogenous TNF-a. J Immunol (1998) 161:2383–90. 9725234

[14] Martinez-Guzman MA Alvarado-Navarro A Pereira-Suarez AL Muñoz-Valle JF Fafutis-Morris M Association between STR -794 CATT5-8 and SNP -173 G/C polymorphisms in the MIF gene and lepromatous leprosy in mestizo patients of western Mexico. Hum. Immunol (2016) 77:985–9. 10.1016/j.humimm.2016.07.006 27426952

[15] Li YX Zhang L Simayi D Zhang N Tao L Yang L Human papillomavirus infection correlates with inflammatory stat3 signaling activity and IL-17 level in patients with colorectal cancer. PLoS One (2015) 10:e0118391. 10.1371/journal.pone.0118391 25706309 PMC4338045

[16] World Health Organisation. World medical association declaration of helsinki. Ethical principles for medical research involving human subjects. J Am Med Assoc (2013) 310:2191–4. 10.1001/jama.2013.281053 24141714

[17] Bansal F Narang T Dogra S Vinay K Chhabra S Serum macrophage migration inhibitory factor levels in leprosy patients with erythema nodosum leprosum. Indian J Dermatol Venereol Leprol (2017). 10.4103/ijdvl.IJDVL 28656911

[18] de Sousa JR Pagliari C de Almeida DSM Barros LFL Carneiro FRO Dias LB Th9 cytokines response and its possible implications in the immunopathogenesis of leprosy. J. Clin. Pathol (2016) 70(6):521–7. 10.1136/jclinpath-2016-204110 27927694

[19] Bernhagen J Calandra T Mitchell R Martin S Tracey K Voelter W MIF is a pituitary-derived cytokine that potentiates lethal endotoxaemia. Nature (1993) 365:756–9. 10.1038/365756a0 8413654

[20] Calandra T Froidevaux C Martin C Roger T. Macrophage migration inhibitory factor and host innate immune defenses against bacterial sepsis. J Infect Dis (2003) 187:S385–90. 10.1086/374752 12792855

[21] Liang Y Yuan W Zhu W Zhu J Lin Q Zou X Macrophage migration inhibitory factor promotes expression of GLUT4 glucose transporter through MEF2 and Zac1 in cardiomyocytes. Metabolism (2015) 64(12):1682–93. 10.1016/j.metabol.2015.09.007 26455966

[22] Brocks T Fedorchenko O Schliermann N Stein A Moll UM Seegobin S Macrophage migration inhibitory factor protects from nonmelanoma epidermal tumors by regulating the number of antigen-presenting cells in skin. FASEB J (2017) 31:526–43. 10.1096/fj.201600860R 27825106 PMC6137604

[23] Salem SA Asaad MK Elsayed SB Sehsah HM. Evaluation of macrophage migration inhibitory factor (MIF) levels in serum and lesional skin of patients with alopecia areata. Int J Dermatol (2016) 55:1357–61. 10.1111/ijd.13344 27420731

[24] Holowka T Castilho TM Garcia AB Sun T McMahon-Pratt D Bucala R. *Leishmania*-encoded orthologs of macrophage migration inhibitory factor regulate host immunity to promote parasite persistence. FASEB J (2016) 30:2249–65. 10.1096/fj.201500189R 26956417 PMC4871794

[25] Hashimoto K Maeda Y Kimura H Suzuki K Masuda A Matsuoka M *Mycobacterium leprae* infection in monocyte-derived dendritic cells and its influence on antigen-presenting function. Infect Immun (2002) 70:5167–76. 10.1128/IAI.70.9.5167-5176.2002 12183567 PMC128241

[26] Lee DJ Sieling PA Ochoa MT Krutzik SR Guo B Hernandez M LILRA2 activation inhibits dendritic cell differentiation and antigen presentation to T cells. J Immunol (2007) 179:8128–36. 10.4049/jimmunol.179.12.8128 18056355 PMC2650749

[27] Landsverk OJB Bakke O Gregers TF. MHC II and the endocytic pathway: regulation by invariant chain. Scand J Immunol (2009) 70:184–93. 10.1111/j.1365-3083.2009.02301.x 19703008

[28] Priscilla V Sens MM Indeterminate leprosy and lepromatous index case: four cases in the same family. An Bras Dermatol (2013) 88:105–8. 10.1590/abd1806-4841.20132050 PMC3875986 24346893

